# Viruses and Tetraspanins: Lessons from Single Molecule Approaches

**DOI:** 10.3390/v6051992

**Published:** 2014-05-05

**Authors:** Selma Dahmane, Eric Rubinstein, Pierre-Emmanuel Milhiet

**Affiliations:** 1 Inserm, Unité 1054, Single Molecule Biophysics Department, Centre de Biochimie Structurale, 34090, Montpellier, France; E-Mail: selma.dahmane@cbs.cnrs.fr; 2 CNRS, UMR 5048, Université de Montpellier, 34090, Montpellier, France; 3 Inserm, U602, 94807, Villejuif, France; E-Mail: eric.rubinstein@inserm.fr; 4 Université Paris 11, Institut André Lwoff, 94807, Villejuif, France

**Keywords:** Tetraspanin, HIV-1, HCV, Single Molecule, Tracking, microdomain

## Abstract

Tetraspanins are four-span membrane proteins that are widely distributed in multi-cellular organisms and involved in several infectious diseases. They have the unique property to form a network of protein-protein interaction within the plasma membrane, due to the lateral associations with one another and with other membrane proteins. Tracking tetraspanins at the single molecule level using fluorescence microscopy has revealed the membrane behavior of the tetraspanins CD9 and CD81 in epithelial cell lines, providing a first dynamic view of this network. Single molecule tracking highlighted that these 2 proteins can freely diffuse within the plasma membrane but can also be trapped, permanently or transiently, in tetraspanin-enriched areas. More recently, a similar strategy has been used to investigate tetraspanin membrane behavior in the context of human immunodeficiency virus type 1 (HIV-1) and hepatitis C virus (HCV) infection. In this review we summarize the main results emphasizing the relationship in terms of membrane partitioning between tetraspanins, some of their partners such as Claudin-1 and EWI-2, and viral proteins during infection. These results will be analyzed in the context of other membrane microdomains, stressing the difference between raft and tetraspanin-enriched microdomains, but also in comparison with virus diffusion at the cell surface. New advanced single molecule techniques that could help to further explore tetraspanin assemblies will be also discussed.

## 1. Introduction

Lateral segregation of membrane components within the plasma membrane of eukaryotic cells is an essential phenomenon for the optimal function of most of biological processes. This includes the diffusion of lipids and proteins within the plasma membrane, as well as their organization into microdomains. It is now well established that plasma membranes of eukaryotic cells are organized as a mosaic of micro or nanodomains that contain specific sets of lipids and proteins (an organization initially proposed by Maxfield [1]). Importantly, recent data based on advanced microscopy techniques suggest that several types of membrane microdomains fluctuate in their composition, due to the permanent exchange of their components with surrounding molecules that diffuse within the membrane (see recent reviews [2,3,4] and the example of tetraspanins below). This type of organization is now well accepted but the size, the stability or life time, and the composition of the different microdomains are still a matter of debate and need to be further explored, e.g., the molecular mechanisms associated to their formation in living cells is poorly documented, even if it is well established that certain lipids, such as cholesterol and sphingolipids, play a key role. Similarly, relationships between the different types of microdomains, such as rafts [5], tetraspanin-enriched microdomains (TEMs), or DC-SIGN (Dendritic Cell-Specific Intercellular adhesion molecule-3-Grabbing Non integrin) microdomains [6], remain unclear. Importantly, membrane components are mainly very dynamic and this dynamics, which is responsible for membrane plasticity and crucial for cell function and survival, needs to be explored in more details.

Membrane microdomains have been associated early on with virus infection (first reviewed in [7]). Characterization of this association was first assessed by co-localizing viral proteins with raft markers using fluorescence microscopy or two techniques classically used to characterize such membrane assemblies, namely the sensitivity of budding and/or egress to cholesterol depletion and the presence of viral components into membrane that resist to Triton X-100 solubilization at 4 °C or DRMs (Detergent Resistant Membrane), the latter being quite controversial with respect to its interpretation [8]. The first characterized example was the influenza virus that was described to be raft-associated by its ability in selecting lipid ordered domains during budding from the apical membrane of epithelial cells [9]. The role of plasma membrane rafts in human immunodeficiency virus type 1 (HIV-1) assembly and release was also described early on [10,11]. HIV-1 incorporates host raft lipids and proteins into its envelope and employs rafts during its life cycle (for a recent review, see [12]). More recently, another type of microdomains has been identified to be important during HIV-1 infection, namely the tetraspanin-enriched microdomains. Their existence within the plasma membrane is related to a network of protein-protein interactions involving tetraspanins and other transmembrane proteins (for more details, see the second section of this review). As in rafts, lipids also play a key role in the membrane organization of tetraspanins, which are palmitoylated and known to interact with cholesterol and gangliosides. However, in contrast to lipid rafts that are typically defined as being detergent insoluble, their interaction networks can be analyzed in the soluble phase of detergent lysates [13].

The initial evidence of the existence of membrane microdomains came from biochemical approaches involving membrane solubilization with detergents and from fixed cells images obtained with both conventional fluorescence and electron microscopy. However, none of these approaches can probe the dynamics of membrane components, a crucial parameter to decipher the molecular mechanisms associated to these microdomains. Thanks to the development of advanced microscopy techniques that break the diffraction barrier, others and we have investigated the dynamics of membrane microdomains at the single molecule level. In this paper, we briefly present single molecule techniques and review the main results concerning tetraspanin and TEMs, especially in the context of virus infection.

## 2. Probing Membrane Organization at the Single Molecule Level

Nowadays, two main approaches in fluorescence microscopy are used to probe membrane dynamics, *i.e.*, ensemble labeling or single molecule techniques (reviewed in [3,14]). In the first case, lateral segregation of membrane components is probed using fluorescence recovery after photobleaching (FRAP). In this technique, an intense focused illumination is used to photobleach fluorophores in a selected region of a specimen. If the fluorescently labeled molecules are mobile, un-bleached molecules will move into the bleached region while the bleached molecules move out. Fluorescence recovery of the photo-bleached region over time can then be used to measure the average diffusion coefficient of a molecular ensemble as well as the percentage of mobile and immobile fractions (reviewed in [15]). However, the spatial resolution is poor because the size of the bleached area often exceeds the diffraction limit. In addition, it is impossible to determine different modes of motion of molecules within the membrane. In the latter case, two main single molecule techniques are currently available, *i.e.*, fluorescence fluctuation spectroscopy (FFS) and single molecule tracking (SMT). FFS is a single-molecule sensitive technique that analyzes fluctuations in the fluorescence emission of small molecular ensembles in a sub-femtoliter volume and thus provides information about a multitude of parameters such as stoichiometry, concentration and molecular diffusion of fluorescently labeled molecules with a very high temporal resolution (reviewed in [16]). Single-point fluctuation correlation methods such as Fluorescence Correlation Spectroscopy (FCS) are local, enabling the determination of diffusion coefficient [16]. Varying the beam area allows the identification of different motion modes [17]. More recently scanning FCS, which consists in moving the illumination volume according to a periodic pattern in the sample, provides additional spatial correlation and it is appealing for the measurement of molecular motion and interaction with single molecule sensitivity. FFS approaches have been used to elucidate important aspects of transmembrane proteins, including interactions of receptors with their ligands and receptor oligomerization (reviewed in [18]) and the lateral heterogeneity of TEMs [19]. However, even if recent conceptual and technical developments in FFS techniques greatly reduce the spatial averaging, they cannot analyze molecules individually precluding the identification of stochatisc events within the plasma membrane of eukaryotic cells.

**Figure 1:**
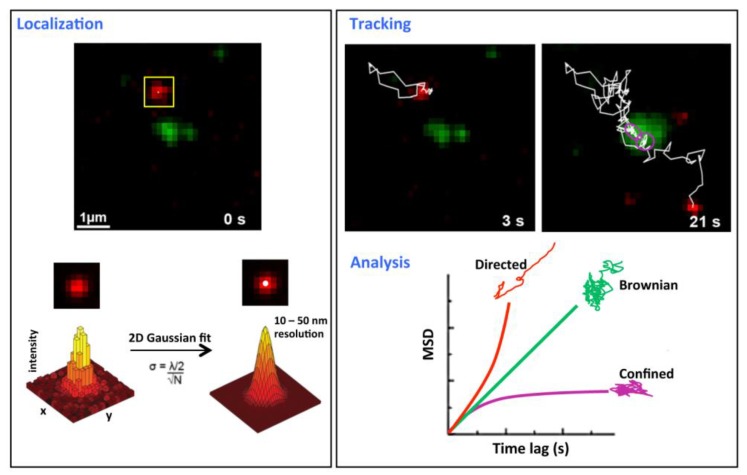
Single molecule tracking principles. This figure describes a tracking experiment of CD9 labeled with Atto647N-conjugated Fab fragments of the anti-CD9 SYB-1 in PC3 cells and recorded with a TIRF microscope equipped with a high numerical aperture objective and a highly sensitive EMCC camera (adapted from [20]). The green labeling corresponds to CD9 ensemble labeling achieved with anti-CD9 Cy3B-labeled antibodies. *Localization* (**left panel**): x-, y- coordinates of single molecules are determined for each frame and are derived from the central position of its diffraction limited intensity profile by applying a 2D Gaussian fit function. The position precision σ is far below the optical resolution and depends on both wavelength (λ) and the number of photons collected (N). *Tracking and analysis* (**right panel**): trajectories are reconstructed frame per frame (white thin line in the upper panel) and analyze by plotting the mean square displacement (MSD) *versus* time lag (lower right panel). The plot is used to classify the type of motion modes for all the trajectories (simplified as Brownian, confined and directed). A linear plot indicates normal diffusion and can be described by <r^2^> = 4DΔt (D, diffusion coefficient) for a two-dimensional analysis (green curve). A quadratic dependence (red curve) indicates directed motion and can be fitted by <r^2^> = v^2^Δt^2^ + 4DΔt (v, mean velocity). When the MSD asymptotically approaches a maximum value for larger Δt (purple curve), the molecule is confined and data can be fitted with MSD (∆t) = (1/3) L^2^ [1 –exp(−12D∆t/L2)] (L^2^ the area of a squared confined region). Purple circles in the tracking frame indicate a transient confined area.

The advantage of SMT as compared to FRAP and FCS is that individual molecules can be analyzed with high spatial and temporal resolution providing a detailed description of their motion within the plasma membrane (Figure 1). SMT is based on the detection of individual molecules tagged with fluorescent markers using sensitive camera such as electron multiplied charge couple device (EMCCD) [21]. Typically, experiments are performed in low-density labeling conditions, resulting in a few fluorescently labeled molecules imaged per frame. Because SMT requires the observation of isolated particles for relatively long periods of time, stable and bright fluorescent markers are required and the most popular fluorescent probes are the cyanin derivatives, such as the cationic Atto647N (see one of the first examples with this probe in [20]) and quantum dots (Qdots). These Qdots are very bright and the most stable probes currently available and can be considered as a tool of choice for tracking proteins within membranes, even though the control of the valency of labeling, an important parameter to control for SMT, is challenging (reviewed in [22]). Of note, tracking can be performed with latex or colloidal gold beads bound to the molecule of interest but the size of the bead, which is about two orders of magnitude larger that the size of the tracked membrane component, could constrain its diffusion (reviewed in [23]). During SMT experiments, videos are collected at high temporal resolution (10–200 frames/s) and the positions of single molecules are located with a precision below 20 nm using computational fitting of the signal intensity profile (Figure 1). Later on, positions of each isolated molecule are linked frame-by-frame and trajectories are reconstructed. The analysis of trajectories is generally based on the plot of the mean square displacement (MSD) as a function of the time lag for characterizing the motion of membrane components. Membrane proteins can adopt different types of motion, namely Brownian, directed or confined diffusion [24]. In addition, a combination of different modes can be observed, *i.e.*, proteins undergoing Brownian motion can be transiently confined, through mechanisms involving interactions with lipids, actin cytoskeleton and/or other proteins. SMT has been used to reveal the lateral organization of membrane proteins such as G Protein-coupled receptors [4,25], GPI-anchored proteins [26,27], tetraspanins [20,28] and to understand the spatial dynamics of molecular interactions in living cells in different cellular contexts. SMT is also an outstanding technique to question whether virus infection is dependent on lateral diffusion and confinement of membrane proteins, whether viruses use and/or alter membrane organization, and to understand the mechanism of viral entry and budding into the host cell.

Similarly to SMT, individual viruses (Influenza, HIV, MLV (Moloney murine leukemia virus), *etc.*) can be tracked in real time, called single particle tracking (SPT) (nicely reviewed in [29]). After they bind to their host cell, SPT allows measuring the apparent diffusion coefficient of viruses within the membrane, before being trapped in specific microdomains or captured by clathrin-coated pits. It has also been possible to follow their internalization, revealing that most of the viruses adopt unidirectional or bidirectional motion toward the nuclear periphery through interaction with actin and microtubules cytoskeleton [30,31,32].

## 3. General Dynamic Behavior of Tetraspanins within Biological Membranes

Tetraspanins are transmembrane proteins involved in many key physiological functions such as immunity [33,34], gamete, macrophage and muscle cell fusion [35,36,37], photoreceptor morphogenesis [38] or cell migration [39]. In agreement with these key roles, tetraspanins are also associated to several pathologies including cancer (recently reviewed in [40]) and infection (developed below). They have the unique property to form with one another and with other transmembrane proteins (integrins, Immunoglobulin superfamily proteins and others) a protein-protein interaction network at the plasma membrane, referred to as the tetraspanin web. Many studies have suggested that tetraspanin functions are linked to this network of interactions between membrane proteins but interaction between tetraspanins and cytoplasmic kinases could also be implicated [41,42,43]. Biochemical analyses of the tetraspanin web using various detergents and cross-linking experiments have revealed its hierarchical nature (reviewed in [44,45]). Thus specific tetraspanins associate with partner proteins to form primary complexes that can further assemble through tetraspanin-tetraspanin interaction and form higher order structures. Examples of primary complexes include CD151 with integrins (e.g., the integrin α_3_ß_1_ or α_6_ß_1_), CD9 or CD81 with EWI-2 or EWI-F/CD9P-1, or Tspan5, Tspan14, Tspan15 with ADAM10 [45,46]. As these complexes partially partition in low-density membrane fractions in sucrose gradients after lysis with mild detergent [13], a technique used to characterize raft microdomains, tetraspanins have been suggested to form membrane microdomains called TEMs or TERMs for tetraspanin-enriched microdomains [47].

Biochemical experiments described above have been and are still necessary to dissect the network of protein-protein interactions but only provide a snapshot of its membrane organization. In this context, SMT was applied to tetraspanins and has first provided a dynamic view of the tetraspanin web [20]. Using TIRF microscopy on prostate cancer cells, we first demonstrated that most of CD9 molecules are dynamic and a majority of them are free to diffuse within the membrane, possibly embedded in small membrane clusters that could accommodate diverse lipids and proteins (in particular, tetraspanins and their partners). Interestingly, thanks to a dual view imaging setup, we also observed that areas enriched in CD9 and its membrane partners, called TEAs for tetraspanin-enriched areas, behave as membrane platforms in permanent exchange with the rest of the membrane (Figure 1). Indeed, diffusing CD9 molecules are transiently trapped in these areas and escape after a time period in the second range. Some CD9 molecules can also be purely confined, at least during the time of observation. CD9 mobility and partitioning are both dependent on its palmitoylation and on the cholesterol content of plasma membrane, underlining the importance of lipids in tetraspanin organization. Interestingly CD55, a GPI-anchored protein resident in raft microdomains, that are biochemically defined by their insolubility in ice-cold non-ionic detergent, was not able to partition in TEAs. The dynamic behavior of CD9 and CD151 was also demonstrated in endothelial cells using FCS [19]. This study highlighted the existence of endothelium adhesive platforms, important in the recruitment of receptors for leukocyte integrin, identified as TEMs but distinct from rafts. More recently, SMT was used to probe the influence of CD151 expression on α_6_ integrin diffusion [48]. This paper showed the propensity of CD151 to modify the membrane behavior of its partners. CD151 expression indeed favored a Brownian or confined diffusion of α_6_ integrin whereas directed diffusion was observed in the absence of the tetraspanin. It was proposed that CD151 promoted recruitment of its partners into TEAs. A similar role was proposed for the tetraspanin CD82 that can restrict the diffusion of EGF receptor [49]. Altogether these first studies revealed the dynamics of several tetraspanins and their potential role as molecular organizers within the plasma membrane. At this point it is important to stress that TEMs are often identified as TEAs. In our view TEAs, as observed in our experiments and characterized using ensemble labeling fluorescence microscopy, should not be confused with TEMs. TEMs are defined as the tetraspanin web and include the entire set of interactions in which tetraspanins are involved, which can occur in and out these TEAs. This view is in agreement with the current definition of raft microdomains, which are described as small, heterogeneous and dynamic nanodomains that can sometimes be stabilized to form larger platforms through protein-protein and protein-lipid interactions [50].

## 4. CD9 and CD81 Partition during Budding of HIV-1 Particles

The role of tetraspanins during infection has been described early on and these proteins are implicated in various aspects of virus life cycle, e.g., many viruses use TEMs to enter host cells and to infect other cells after their assembly and budding (see reviews [51,52,53]). Using SMT, we have tried to further understand both organization and modulation of the tetraspanin web during the budding of HIV-1 and the entry of Hepatitis C virus (HCV).

Human immunodeficiency virus (HIV) is a major cause of morbidity and mortality around the world. There are two types of HIV (HIV-1 and HIV-2) but, worldwide, the predominant virus is HIV-1 and it is probably one of the most studied pathogens. One of the critical steps of HIV-1 life and reproduction cycle is its assembly and the release of new virions from infected cells [54]. It is known that HIV-1 buds specifically from the plasma membrane [55] and this process involves the polyprotein Gag that consists of five subdomains. Interestingly the expression of Gag alone is sufficient to induce the formation of virus-like particles (VLP) from the membrane of many cell lines, thus affording a convenient approach to explore the mechanism of HIV-1 virus egress [56]. Using such particles containing fluorescently labeled Gag proteins, the dynamics of VLP formation has been studied and it was shown that HIV-1 budding occurs in three phases, with an average duration from assembly to release of approximately 30 min [57,58]. Phase 1 correlates with the targeting and attachment of Gag proteins to the inner leaflet of the plasma membrane through its MA domain (amino terminal myristoylated matrix domain). After Gag multimerization at the assembly sites in a few minutes, the ESCRT (Endosomal Sorting Complex Required for Transport) machinery of the host cell is recruited, through the P6 domain of Gag proteins (phase 2), allowing the release of new VLPs (phase 3). These three phases require a precise and coordinated recruitment of membrane components, both lipids and proteins, in time and in space. It was proposed that some events appear as primarily protein-driven whereas others are lipid-dependent [59]. The later assumption came from the implication of lipid rafts in the early phase of HIV-1 budding. Indeed HIV-1 membrane is enriched in cholesterol and GM3 [60] and cholesterol depletion blocks HIV-1 release [61]. In addition, phosphatidyl-inositol-(4,5)-bisphosphate (PI(4,5)P2) appears to be an important determinant for Gag localization at the plasma membrane (reviewed in [62]). Yet the membrane landscape is more complicated since additional microdomains have been associated to HIV-1 replication cycle. In this regards, several tetraspanins (CD9, CD81 and CD63) accumulate in Gag-enriched areas mostly corresponding to budding sites in T cells [63,64] and in HeLa cells [65]. In the latter case, tetraspanins also co-localize with the HIV-1 envelope glycoprotein and with two components of the mammalian ESCRT1 machinery, TSG101 and VPS28. In addition, it has been shown that expression of tetraspanins can alter HIV-1 progression and that these tetraspanins are incorporated in the membrane of virus particles [66,67]. In agreement with the involvement of CD9 and CD81 in several fusion processes, tetraspanins were proposed to play an important role in membrane fusion induced by HIV-1 envelope [66]. In addition to its presence in TEMs at the plasma membrane, CD63 also plays an early post-entry role prior to or at the reverse transcription step [68].

**Figure 2:**
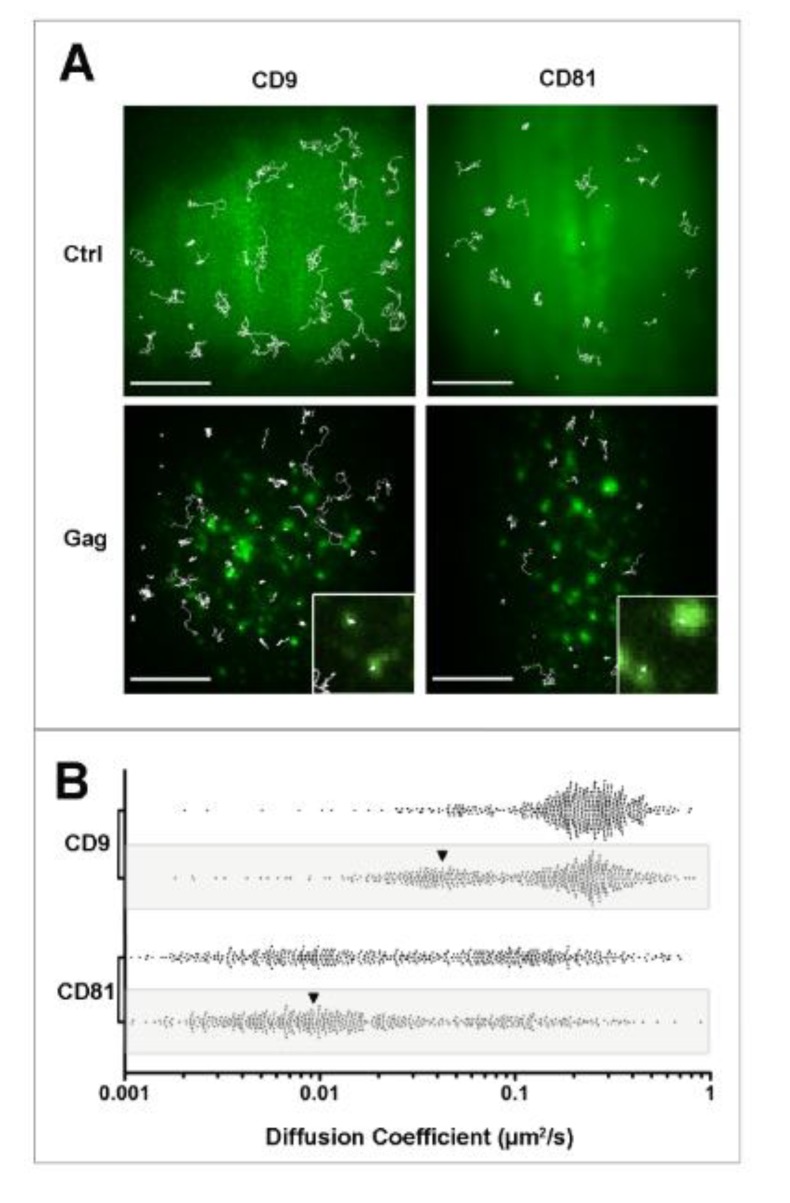
Single molecule analysis of CD9 and CD81 behavior during HIV-1 budding. Panel A: Micrographs showing the superimposition of ensemble labeling of GFP (control) or Gag fused to GFP (Gag) fluorescence signal with several representative single molecule trajectories (white lines) obtained after tracking Atto647N-conjugated Fab fragments of anti-CD9 or anti-CD81 antibodies in HeLa cells. Upon gag expression, dynamics of both tetraspanins was decreased, quantitated in panel B, mainly due to their confinement. The insets correspond to a zoom where confinements of CD9 and CD81 in Gag-enriched areas are observed. A larger effect was observed for CD9 because it is much more dynamics than CD81 under native conditions (see control panels and scatter plots in B). Scale bars represent 10 µM. Panel B shows scatter plots representing the distribution of all the apparent diffusion coefficients of CD9 and CD8, calculated from the MSD-time lag plot in control or in Gag-expressing (grey box) cells. Each dot represents one trajectory (500 trajectories are shown here). The black arrowheads highlight the increase in confined trajectories (adapted from [69]).

All these publications emphasized the importance of tetraspanins in HIV-1 budding but the influence of Gag assembly on tetraspanin organization were still unexplored. We therefore investigated the dynamics of CD9 and CD81 by combining both SMT and FRAP in HeLa cells expressing GFP-tagged Gag proteins [69]. We initially demonstrated using FRAP experiments the existence of progressive CD9 enrichment at Gag assembly sites, suggesting that Gag induces CD9 trapping. Indeed, the mobile fraction (mf) of Fab-labeled CD9 molecules revealed that CD9 molecules are significantly less mobile in Gag expressing cells (mf 29%) in comparison to control cells (mf 76%). In addition, CD9-enriched areas containing Gag puncta within the bleached area failed to recover to their initial intensity, whereas areas adjacent to Gag puncta recovered most of their CD9 signal. Interestingly no difference was observed for the ganglioside GM1 that is often used as a raft marker. To further support these observations, CD9 and CD81 single molecules were tracked using Atto647-labeled Fab fragments with a high temporal (100 ms) and spatial (50 nm) resolution using TIRF microscopy. Interestingly we have observed a specific confinement of the two proteins in Gag-enriched areas (Figure 2). In fact, apparent diffusion coefficient of CD9 and CD81 molecules both decreased in Gag-expressing cells compared to control cells (from 0.24 ± 0.03 to 0.16 ± 0.03 µm^2^/s for CD9, values comparable with diffusion coefficients measured by FRAP). The decrease in CD9 dynamics was attributed to a confinement of tetraspanins during Gag assembly, the percentage of CD9 molecules exhibiting confined motion increasing significantly from 14% to 34% of the total number of trajectories, at the expense of Brownian trajectories (from 48% to 27%). Similarly, confinement of CD81 molecules strongly increased from 20% to 42% of the total number of trajectories. Surprisingly, CD81 dynamics in HeLa cells was completely different from that of CD9 (CD81 is much more confined than CD9), even if the two molecules are often co-localized and share membrane partners. This difference can be in part explained by CD81 interaction with the cytoskeleton (manuscript in preparation). In the set of experiments described above, the complexity of membrane organization required control proteins to validate our observations. CD46, a protein that is not localized in lipid rafts and minimally associates with TEMs but which has been identified in HIV-1 particles, was used as a control and its membrane behavior was mostly unaffected upon Gag expression. The stochasticity of the recorded events also requires getting a high number of trajectories to confidently describe the behavior of each protein. Typically, using our experimental conditions, we calculated that a minimum of 300 trajectories was necessary to be statistically significant.

As raft microdomains had been associated to HIV-1 budding [62] and because of the importance of lipids in both organization and function of the tetraspanin web and rafts, it was important to address the specificity of tetraspanin recruitment by Gag as compared to a raft marker. Ensemble labeling of the GPI-anchored protein CD55 mentioned above in non-fixed cells showed an overlapping of the raft marker with Gag proteins, similarly to what was observed with tetraspanins. This result is in good agreement with the paper of Ono’s group describing the coalescence of clustered lipid rafts and TEMs induced by Gag targeting to the plasma membrane, even if in this case, clustering was artificially induced by antibody-mediated copatching used to sharpen fluorescence signals obtained for the raft marker CD59 and tetraspanins CD9 and CD81 [70]. At the single molecule level, CD55 membrane behavior was also altered upon Gag assembly but to a lesser extent as compared to the tetraspanins CD9 and CD81 (the increase of confinement induced upon Gag assembly was more limited). Single molecule analysis therefore appears as a very powerful tool to discriminate between different membrane behaviors that cannot be probed using ensemble labeling and our results emphasize that TEM and raft are different entities (see also [19,20]). Such assumption is also supported by cholesterol oxidase treatment of HeLa cells showing that CD55 dynamics in Gag-expressing cells is much more sensitive than CD9 to cholesterol depletion, both at the ensemble or single molecule level (the increase in CD55 confinement by Gag was almost completely abolished by cholesterol oxidase treatment). Membrane dynamics measured by FRAP also supported differences between the raft component GM1 and CD9 [69]. Taken together, our single molecule experiments demonstrated that the tetraspanins CD9, CD81, and to a lesser extent CD55 are specifically recruited into membrane areas where Gag multimerizes and that Gag is not targeted to preformed raft or tetraspanin membrane microdomains as proposed before. However, we cannot exclude that Gag multimerizes into another type of preformed microdomain. This specific recruitment of CD9 and CD81 at Gag assembly sites could explain why these tetraspanins are enriched in the viral membrane as compared to other components of the plasma membrane of host cells. SMT experiments also highlight both complexity and specificity of the mechanisms of membrane proteins recruitment during HIV-1 budding.

## 5. Membrane Behavior of CD81, a Key Molecule in HCV Entry

While several tetraspanins are involved in HIV-1 infection, CD81 plays a specific and essential role in the entry in hepatocytes of two major human pathogens, the malaria parasite *Plasmodium* [71] and the Hepatitis C virus (HCV) [72]. In the latter case, CD81 has been identified as an essential HCV receptor. This human pathogen infects hepatocytes leading to progressive liver disease including fibrosis, cirrhosis and hepatocellular carcinoma and is currently an important problem of public health. Determining the molecular mechanisms associated to virus entry is therefore very important.

HCV entry is a complex phenomenon requiring virus binding to several transmembrane proteins (recently reviewed in [73,74] and in Feneant *et al.* in this special issue). HCV first interacts with attachment factors such as glycosaminoglycans and the Low Density Lipoproteins Receptor (LDL-R). After this first attachment step, viral particles interact with a series of entry factors including the scavenger receptor SR-BI, CD81 and two tight junction proteins, Claudin1 (CLDN1) and Occludin. Importantly, CD81 and CLDN1 associate to form functional complexes, which are essential to HCV entry [75]. Moreover, this membrane partnership is likely involved in HCV internalization via a clathrin- and dynamin-dependent process [76]. CD81 especially plays a major role in HCV entry through its direct interaction with the E2 envelope glycoprotein exposed at the surface of HCV virion [72] and numerous studies have shown that cell susceptibility to HCV infection is closely related to the CD81 expression level (reviewed in [77,78]). HCV was also shown to enter Huh-7 hepatocytes in membrane areas enriched in CD81 [79]. In addition, the facilitation of HCV entry by palmitoylated CD81 that preferentially associates with TEMs underlines the key role of these microdomains [80]. Lipids also appear to play a key role in HCV entry which is blocked when HuH-7 cells are treated with sphingomyelinase, an enzyme decreasing the sphingomyelin content within the plasma membrane at the profit of ceramides [81]. In addition, related with the involvement of lipids is the restriction of HCV entry when HepG2 hepatoma cells are polarized [82]. Indeed, it is known that lipid composition is different between apical and basolateral membranes [5] and that polarization likely induces important changes in partition of membrane components that could influence TEMs. Finally, membrane partnership of tetraspanins with primary partners, within or outside TEMs, appears very important for HCV entry. Indeed expression of the CD81’s partner EWI-2wint, a cleavage product of EWI-2, inhibits HCV entry by preventing interaction between the HCV envelope glycoprotein E2 and CD81 [83].

**Figure 3:**
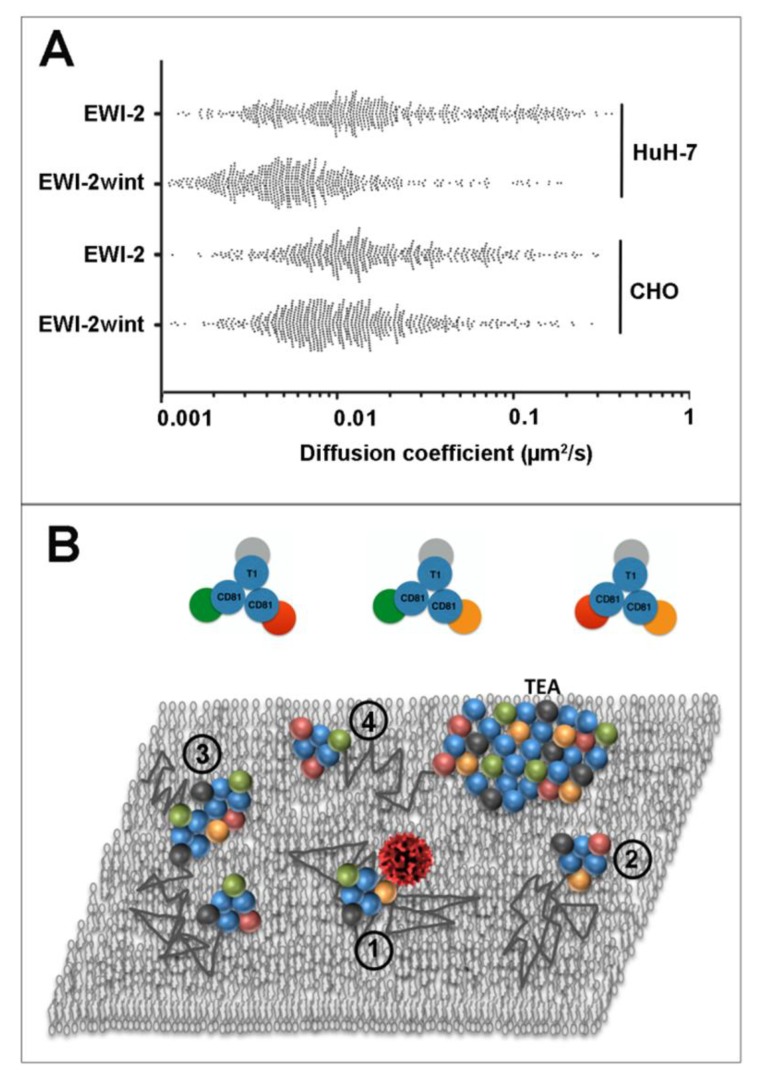
EWI-2wint decreases CD81 dynamics in hepatocytes. (**A**) represents the distribution of all the apparent diffusion coefficients calculated for CD81, in Huh-7 clones expressing EWI-2 or EWI-2wint or in CHO cells transfected with human CD81 in combination with either a non-cleavable EWI-2 construct (EWI-2) or WT EWI-2. In CHO cells, EWI-2 is cleaved to produce EWI-2wint (adapted from [85].) Cells were labeled with Atto647N-labelled Fab fragments of TS81. Each dot represents one trajectory and 500 trajectories are represented for each condition. (**B**) is a simplistic view of tetraspanin organization in the plasma membrane, together with some key partners involved during HCV infection (EWI-2 in orange, EWI-2wint in red, CLDN1 in green, an unknown primary partner in grey; tetraspanins are in blue and their names indicated; T1 could be any tetraspanin). A virus is schematized in red, the lipid matrix is in grey, and the dark thin lines are trajectories of diffusing clusters, composed of tetraspanins and partners, which can sometimes interact with each other (some possible clusters are shown in the upper part). Different situations are depicted in the model of plasma membrane: (1) corresponds to a cluster suitable for infection; in (2), the cluster is devoid of CLDN1 and is not competent for virus internalization as well as the larger complex; in (3) formed thanks to EWI-2/EWI-2wint dimerization; EWI-2wint could also favor confinement of CD81 and CLDN1 through its association to TEA (4). In this model, we assume that the diffusion coefficient is proportional to the size of the clusters.

In order to elucidate the protein/protein/lipid orchestration during HCV entry, we have used SMT to analyze CD81 membrane behavior in hepatocytes, in combination with biochemical approaches, and demonstrated that restricting CD81 diffusion impaired the infectivity of the virus [84,85]. In a first study performed in collaboration with McKeating’s group, we combined SMT and FRAP to probe CD81 dynamics in polarized HepG2 cells. We observed a decrease in the dynamics of proteins localized on the membrane facing the growth substrate and corresponding to the basolateral compartment of polarized cells [84]. As observed in other cell types, CD81 motion comprises two modes of diffusion, Brownian and confined, or a combination of these two modes referred to as mixed trajectories. We demonstrated that polarization clearly favored confinement of the tetraspanin at the basal surface of the cell. This decrease was due to a dual effect: (i) an increase of CD81 transient confinement in mixed trajectories; and (ii) a lower diffusion coefficient of molecules displaying a Brownian behavior (from 0.17 to 0.11 µm^2^/s). The latter observation can be explained by a decrease of membrane fluidity since a similar decrease was observed with a lipid marker (from 0.45 to 0.17 µm^2^/s). A lower percentage of cholesterol in the basolateral membrane following polarization could explain this result, taken into account that this lipid is implicated in the apical targeting of GPI-anchored proteins [86]. Similarly, cholesterol is a key component of the tetraspanin network [87] and modification of its membrane content should lead to perturbation of the organization of tetraspanin assemblies. This assumption is in good agreement with the decrease of CD9 and CD81 dynamics observed when cholesterol is depleted in HeLa cells ([20] and data not shown). Interestingly, the membrane diffusion of lentiviral particles pseudotyped with HCV glycoproteins (HCVpp) was shown to be in the same range as the one of CD81. These results strongly suggest that HCV and CD81 co-diffuse within the membrane, even if double tracking experiments need to be performed to confirm this assumption. Taken together, this work suggests that CD81 needs to freely diffuse within the plasma membrane, to interact with membrane partners such as CLDN1 (see the model in Figure 3), and that this free diffusion, impaired by polarization, is required for HCV entry.

A similar conclusion for the role of CD81 dynamics in HCV entry was brought by another study performed in collaboration with Cocquerel’s group [85]. As mentioned above, EWI-2wint is a cleavage product of EW2 that inhibits HCV entry. Surprisingly, CD81 diffusion, measured by SMT in hepatic Huh-7 cells, was impaired in cells expressing EWI-2wint, due to an increase in confinement of CD81 in CD81-enriched areas (Figure 3). It is thus tempting to speculate that this increase is caused by CD81 being trapped within membrane domains, e.g., the stabilization of CD81 in membrane platforms enriched in tetraspanins and their partners. Even if molecular mechanisms associated to this phenomenon need to be elucidated, a stronger affinity of EWI-2wint for CD81 as compared to EWI-2 could explain our results. Alternatively heterodimers EWI-2/EWI-2wint, described in [88], could favor the stabilization of larger complexes. EWI-2/EWI-2wint heterodimerization could also explain the other major effect of EWI-2wint expression, namely the decrease of the diffusion coefficient of CD81 that freely diffused within the plasma membrane (from 3.1 to 1.9 × 10^−2^ µm^2^/s as compared to control cells). Even if we cannot completely exclude that the lipid composition is modified upon EWI-2wint expression, the obvious explanation is that CD81 molecules diffused in larger nanoclusters (Figure 3), assuming that the diffusion coefficient of a molecule is proportional to its size [89]. A preferential role of protein-protein interactions rather than lipid composition in the effect of EWI-2wint expression is also supported by the fact that CD9 dynamics, used as a control, was not modified in Huh-7 cells expressing EWI-2wint. In a functional point of view, as suggested in our study with polarized HepG2 cells [84], restriction of CD81 dynamics could impair CD81/CLDN1 membrane partnership, either by dissociating CLDN1 from CD81-containing dynamic nanoclusters or by sequestrating Claudin-1 in TEAs then preventing HCV entry. The increase of CD81/CLDN1 co-localization in EWI-2wint-expressing cells is in favor of the later explanation. Taken together, these two studies highlight the subtle regulation in time and space of the tetraspanin network of interaction. As observed for CD9 in HeLa and PC3 cells [20,69], CD81 membrane behavior is also cell-dependent, CD81 appearing much slower in Huh-7 than in HepG2 cells. Such a difference could be explained by the level of expression of this molecule within the plasma membrane, the density of the protein playing a role in partnerships of tetraspanin with each other and with their primary partners. Dynamics of the different co-receptors (CLDN1 and SR-BI) now need to be compared one with another in order to decipher the molecular mechanisms associated with HCV entry. In this context, dual color SMT experiments should be very helpful. In addition to the dynamic view provided by SMT, we believe that a precise molecular mapping of CD81 with its membrane partners will be very helpful (see the general perspectives below).

## 6. Conclusions and Perspectives

Among the various functions in which tetraspanins play a role, several implicate membrane remodeling and/or curvature, such as gamete fusion, endocytosis, exocytosis, and both entry and exit of viruses during host cell infection. In addition a relationship between these molecules and tubular membrane structures has been proposed [90,91]. Tetraspanins are also considered as scaffolding proteins due to their ability to form, in a specific lipid environment, a web of interactions with many essential proteins including some bound to the actin cytoskeleton through their direct association with ezrin-radixin-moesin (ERM) proteins [92]. Similarly to numerous other membrane proteins, tetraspanins are dynamic molecules that freely diffuse within the plasma membrane, probably as clusters composed of several proteins (see the model in Figure 3), but can also be confined in TEAs for various periods of time. However, the link between tetraspanin dynamics and function remains unknown. How the size of these membrane assemblies is regulated or whether TEAs are a reservoir of functional proteins are questions that remain to be addressed.

The results presented in this review and based on SMT experiments clearly indicate that, upon viral infection, the general membrane motion (Brownian, confined and transiently confined) of these proteins is indeed altered but so far we have not been able to properly identify the molecular mechanisms behind this alteration because of the complexity of the membrane landscape. Further experiments to better understand how the different partners interact with each other are clearly needed, from the membrane behavior of each partner to their dynamic interaction. In the latter case, two-colors SMT should be very helpful, as well as FCCS (Fluorescence Cross Correlation Spectroscopy) that allow dynamic probing of protein-protein interaction at different time scales. Even if several papers have stressed on the differences between TEMs and rafts, comparing tetraspanins with raft markers such as GPI-anchored proteins is mandatory to understand structure-function relationship within plasma membranes. The importance of the lipids such as cholesterol should also be deeply investigated in regards to their key role in the formation of microdomains.

Besides the dynamics of tetraspanin assemblies or microdomains, their composition and stoichiometry are still poorly documented and we believe that recently developed super resolution techniques, that improve 10 fold the resolution of optical microscopy, should be very helpful. For example, single molecule localization microscopies like Stochastic Optical Reconstruction Microscopy (STORM) [93] or Photoactivated Localization Microscopy (PALM) [94] have already been successfully used to study viruses as well as the distribution of membrane components in a viral context, thereby offering an effective visualization of molecules with a lateral resolution down to 40 nm. Recruitment of HIV-1 proteins (Gag and Env) and host proteins at the plasma membrane has been investigated with this approach [95,96,97]. More recently, CD81 mapping was performed using STORM imaging in a study describing the importance of the tetraspanin in uncoating and budding of Influenza virus [98]. As mentioned above, tetraspanin functions are often associated to membrane curvature and it will be essential to map tetraspanin taking into account cell topography. Hence, combining super resolution imaging with atomic force microscopy should provide crucial information about the mechanisms of HIV-1 and HCV virus entry. Such a combination has been successfully exploited to analyze RNA polymerase packaging in vesicular stomatitis virus [99] and we are currently mapping CD9 and CD81 in the context of HIV-1 budding (work in progress). In relation with membrane topology, z position of single molecules in fluorescence microscopy is also an essential parameter to completely describe the membrane landscape in the infection context. New developments in 3D tracking and 3D super resolution microscopy will be very helpful.
